# The implications of the glenoid angles and rotator cuff status in patients with osteoarthritis undergoing shoulder arthroplasty

**DOI:** 10.1186/s12891-020-03690-8

**Published:** 2020-10-09

**Authors:** Omer Ozel, Robert Hudek, Mohamed S. Abdrabou, Birgit S. Werner, Frank Gohlke

**Affiliations:** 1 Department of Orthopaedics, Baskent University Istanbul Hospital, Oymacı sok, no:7 34662 Altunizade Uskudar, Istanbul, Turkey; 2 Department of Shoulder Surgery, Rhön Klinikum, Bad Neustadt an der Saale, Germany

**Keywords:** Shoulder arthritis, Rotator cuff, Glenoid version angles, Glenoid inclination angles, 3D measurements, Preoperative planning

## Abstract

**Background:**

The success of shoulder arthroplasty, both reverse and anatomical, depends on correcting the underlying glenoid deformity especially in patients with an osteoarthritis. We hypothesized that the distribution of glenoid version and especially inclination are underestimated in the shoulder arthritis population, and also that superior glenoid inclination can be detected through 3-dimensional (3D) software program of computed tomography (CT) to a greater proportion in patients with rotator cuff insufficiency, but also in patients with osteoarthritis with an intact rotator cuff. Because of the influence of rotator cuff imbalance on secondary glenoid wear the values of the critical shoulder angle (CSA) and the fatty infiltration of the rotator cuff are further analyzed. The aim of our study is to determine; 1) the distribution of glenoid inclination and version; 2) the relationship between glenoid inclination, version, the critical shoulder angle (CSA) to the status of the rotator cuff; 3) the proportion of patients with both an intact rotator cuff and a superior inclination greater than 10°.

**Methods:**

A total of 231 shoulders were evaluated with X-ray images, 3-dimentional (3D) software program of computed tomography (CT), and magnetic resonance imaging. The cohort was divided into 3 groups according to their inclination angles and also grouped as intact-rotator cuff and torn-cuff group.

**Results:**

The median (min/max) values for the 231 shoulders were 8° (− 23°/56°) for the inclination angle, − 11°(− 55°/23°) for the version angle, and 31.5°(17.6°/61.6°) for the CSA. The majority of the glenoids were found to show posterior-superior erosion. Glenoid inclination angle and CSA were significantly higher in torn-cuff group when compared with intact-cuff group (*P* < 0.001, both). The rotator cuff tears were statistically significant in high inclination group than low inclination group and no inclination group (*p* < 0.001). In the high inclination group, 41 of 105 (39%) shoulders had an intact rotator cuff, in about 18% of all shoulders.

**Conclusion:**

Our findings show that 3D evaluation of glenoid inclination is mandatory for preoperative planning of shoulder replacement in order to properly assess superior inclination and that reverse shoulder arthroplasty may be considered more frequently than as previously expected, even when the rotator cuff is intact.

**Level of evidence:**

Level III.

## Background

The success of shoulder arthroplasty, both reverse and anatomical, depends on correcting the underlying glenoid deformity [1–3]. The Walch classification, based on the wear pattern and two-dimensional (2D) measurements of retroversion of the glenoid and the amount of posterior subluxation of the humerus, is the most commonly used classification of glenohumeral pathology in patients with primary osteoarthritis [4]. However, the glenoid inclination angle has also been proposed as an important characteristic of glenoid pathology because this angle may be associated with rotator cuff tears, as well as with superior migration of the humeral head [5–7].

Another commonly used characteristic of the glenoid, the critical shoulder angle (CSA) defined by Moor, seems to be closely related to glenoid inclination [5, 8]. Several studies have proposed that either the glenoid inclination or the CSA predisposes to rotator cuff tears and might cause superior migration of the humeral head [6, 8–10]. .If so, both coronal and axial deformities of the glenoid should be corrected in total shoulder arthroplasty. Guidelines for selecting implant design have been established, as have 2D-classifications of glenoid deformities: the transversal plane by Walch et al. [4] and in the coronal plane by Sirveaux and Favard [11].

A normal intrinsic glenoid inclination angle, as measured on reformatted computed tomography (CT) scans, is generally between 0° and 10° [12]. Studies of the retroversion of the glenoid in osteoarthritic shoulders have reported normal values of 2° to 4°; a retroversion of 10° or greater is commonly corrected to avoid overloading the glenoid components and to prevent early loosening of anatomical shoulder arthroplasty [13, 14].

Glenoid version and inclination angles can be measured with different methods [1]. Recently, automated 3D-planning software systems have proven to be as reliable and more accurate than 2D-systems for measuring glenoid version and inclination angles [13, 15, 16]. However, few studies have evaluated automated 3D-planning software measurements of glenoid deformity in the arthritic shoulder and its relationship to the status of the rotator cuff, especially regarding deviation of glenoid inclination. We feel that a significant deviation of glenoid inclination and version can be the result of both a pre-existing anomaly and secondary glenoid wear and we assume that the direction is mainly influenced by the muscle forces especially the imbalance following rotator cuff insufficiency. However, with intact rotator cuff tendons and fatty infiltration of muscles Goutallier classification degree less than 3, we expected superior inclination exceeding 10° in a certain, hitherto unknown proportion. Furthermore, we were interested in evaluating the relationship of CSA as a preexisting anatomical variation in these cases.

Thus, in patients with primary and secondary osteoarthritis and, within the latter, rotator cuff arthropathy of the shoulder joint, we sought to determine 1) the typical degree of glenoid version and inclination angles, as well as the most common direction of glenoid erosion and distribution of the arthritic glenoid angles; 2) the relationship between glenoid inclination, version, the CSA and, the status of the rotator cuff to the glenoid inclination angles; and, especially 3) the proportion of patients with osteoarthritis having both an intact rotator cuff and a superior inclination greater than 10°.

## Methods

We retrospectively evaluated the preoperative characteristics of patients with primary and secondary osteoarthritis and rotator cuff arthropathy scheduled for primary shoulder replacement at our institution between March 2015 and March 2018. Patients with proximal humerus fractures, fracture sequelae, revision surgery, and chronic or neglected shoulder dislocation were excluded. All patients were evaluated with standard anterior-posterior (AP) radiographs, CT scans reformatted in the scapular plane, and with the validated preoperative Glenosys automated 3D software planning program (Imascap, Brest, France) [17]. Magnetic resonance imaging (MRI) was used in 43 patients for further preoperative evaluation of rotator cuff pathology, especially when we expected that CT images did not always differentiate properly between fibrosis, fat accumulation and muscle atrophy. Additionally, the status of rotator cuff tendons and size of tears were evaluated and noted intraoperatively. The CSA was measured with Moor’s technique [8] from standard AP radiographs with the arm in the neutral position. The automated 3D software program generated scatter plots of the distribution of glenoid version and inclination angles for all shoulders and for those with and without intact rotator cuffs.

The direction of glenoid erosion was determined on 3D reconstructions of CT scans, and the inclination and version angles were determined with the automated 3D software program. The transition line between the neoglenoid and the paleoglenoid in cases of glenoid wear were determined in the 3D reconstruction and glenoid wear was grouped to the 2D classifications for primary osteoarthritis according to Walch [4] and in cuff tear arthropathy cases according to Sirveaux and Favard [11]. The direction (perpendicular to the transition line) of glenoid wearing area was graphed to the orthogonal coordinate system. The direction of the glenoid erosion was categorized as postero-superior, posterior, postero-inferior, and antero-inferior. The allocation to the 2D classifications of Walch et al. additionally, Sirveaux and Favard was performed by two independent observers with Walch [4] and Favard [12] using standard transversal and coronal standard planes of the CT scans.

Patients were divided into three groups according to the size of the superior glenoid inclination angle: those with an inclination angle of 10° or more (the high-inclination group), those with an angle between 0° and 9° (the low-inclination group), and those with an angle of 0° or less (the no-inclination group).

To evaluate the status of the rotator cuff regarding tendon tears, muscle atrophy and fatty infiltration according to Goutallier et al [18]. were evaluated thru CT scans for all, and in addition 43 patients with MRI’s. Further, rotator cuff status was noted intraoperatively, especially regarding the size of full thickness rotator cuff tears that involved one or more tendons. The allocation to groups was created according to CT, MRI, and surgical reports. Patients were divided into two groups, one with intact rotator cuffs (the intact-cuff group) and one with full thickness rotator cuff tears of at least the supraspinatus tendon (the “torn-cuff” group). Rotator cuff arthropathy related to the findings in standard AP radiographs were classified according to Hamada et al. [19] grade 1–5. Because of the difficult differentiation between rotator cuff tear arthropathy Hamada 4 and 5 and other types of secondary osteoarthritis including rheumatoid arthritis and late stage crystal-related OA, we did not split our evaluation between the patients within the “torn-cuff” group.

### Statistical methods

All data were analyzed with the Statistical Package for the Social Sciences (SPSS) software (version 17.0, SPSS IncChicago, IL, USA). All continuous variables are expressed as mean ± standard deviation, if they show normal distribution (*p* > 0.05 in Kolmogorov-Smirnov test). All continuous variables are expressed as median, if they show non-parametric distribution. All categorical variables are defined as frequency and percentage. The categorical variables between the groups were analyzed by using the Chi square test or Fisher Exc. test. We compared the inclination angles and CSA values by inclination group (high, low, and no) and by rotator cuff status (intact or torn) with Mann Whitney U test and Student T tests. Correlations between the inclination angle, the version angle, and the CSA were assessed with Spearman’s Correlation. The level for statistical significance was predetermined at *p* < 0.05. Cronbach’s alpha reliability and interobserver correlation were evaluated among the researchers to classification, measurement of glenoid erosion, inclination and version angles of glenoid. Cronbach’s alpha was 0.96; the inter-observer correlation coefficient was 0.94 (95% CI 0.91–0.99).

## Results

The 202 eligible patients (mean [min-max] age, 70 [35–90] years) provided a sample of 231 shoulders; 144 (62%) female shoulders and 87 (38%) male shoulders. Of the 231 shoulders, 7 had avascular necrosis, 13 had rheumatoid arthritis, 111 had primary osteoarthritis and, 100 patients suffered from secondary OA with rotator cuff deficiency (e.g. cuff arthropathy). Of the latter only 27 were rated as Hamada stage 4 and stage 5.

**Fig. 1:**
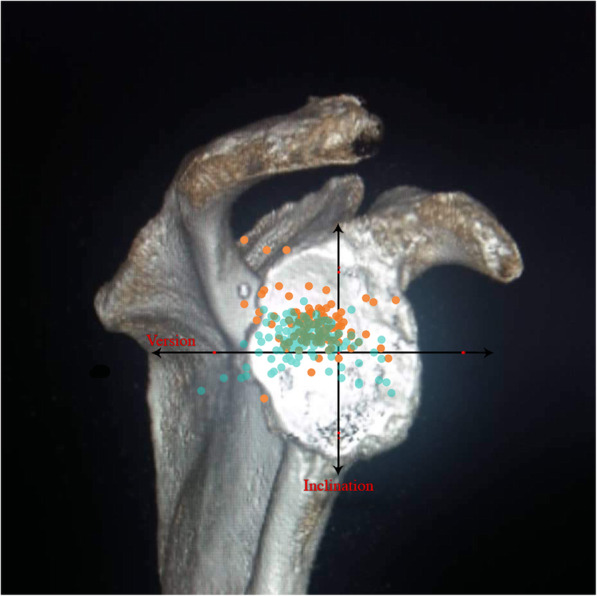
The distribution of glenoid inclination and version angle of the cohort

**Fig. 2:**
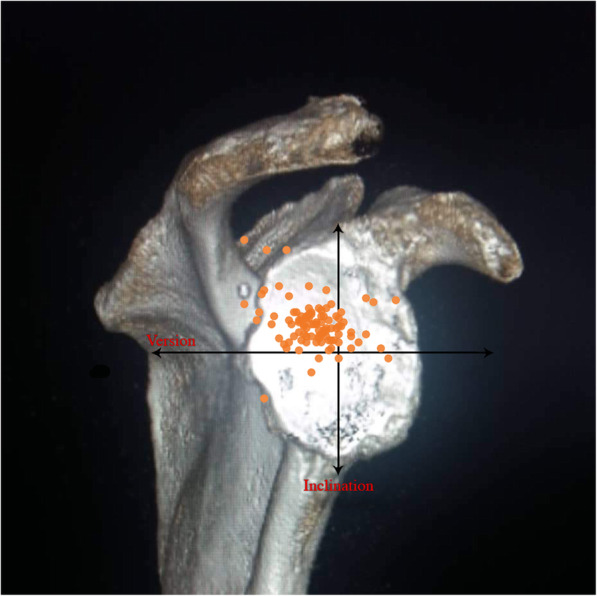
The distribution of glenoid inclination and version angle for the torn-cuff group

**Fig. 3:**
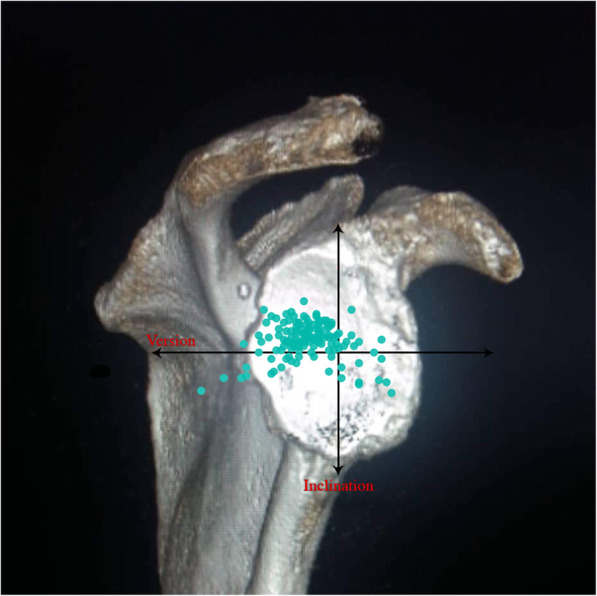
The distribution of glenoid inclination and version angle for the intact-cuff group

Median (min/max) values for the 231 shoulders were 8° (− 23°/56°) for the inclination angle, − 11° (− 55°/23°) for the version angle, and 31.5° (17.6°/61.6°) for the CSA (Table 1). The glenoid inclination angle was significantly correlated with the CSA (*r =* 0.55, *P* < 0.001) and insignificantly with glenoid version (*r =* − 0,08, *P* = 0.2). Glenoid version angle was not correlated with CSA (*r =* − 0.05, *P* = 0.41; Table 1). Of the 231 shoulders of osteoarthritis 35 (15.1%) were classified as Walch B2 (biconcave posterior wear), 19 (8.2%) as B3 and, 13 (5.6%) cases of the rotator cuff arthropathy cases with biconcave wear were allocated to the E2 type, 20 (8.6%) to E3 according to Sirveaux and Favard. (Table 2). The rotator cuff muscles fatty infiltration according to Goutallier classification given in Table 3. Because some cases could be rated both in the axial 2D CT planes as B2 or B3 and in the Sirveaux-Favard classification as E2 or E3, the numbers in Table 2 exceed the total number of all cases. Most of the shoulders with torn rotator cuffs showed posterior-superior direction of glenoid erosion (*n* = 69, 69%; Table 1). Glenoid inclination angle and CSA were statistically significantly higher in torn-cuff group when compared with intact-cuff group (*P* < 0.001, both; Table 1). Glenoid inclination angle was correlated with rotator cuff tears (*r =* 0.44, *P <* 0.001) and with CSA (*r =* 0.55, *p <* 0.001). CSA was also correlated with rotator cuff tears (*r =* 0.61, *p <* 0.001). The distribution of glenoid inclination and version angle of the cohort was given at Fig. 1. The distribution of glenoid inclination and version angle for the torn- cuff group and the intact-cuff group were given at Figs. 2 and 3.

**Table 1:** Preoperative Direction of Glenoid Erosion Patterns and Joint Angles for 231 Shoulders from 202 patients with Osteoarthritis Undergoing Shoulder Replacement

Category	All shoulders (*N* = 231)	Intact rotator cuff (*n* = 131)	Torn rotator cuff (*n* = 100)
Direction of glenoid erosion, n (%)^a^			
Posterior-superior	116 (50.2)	47 (35.8)	69 (69)
Posterior	102 (44.2)	73 (55.7)	29 (29)
Posterior-inferior	11 (4.7)	9 (6.8)	2 (2)
Anterior-inferior	2 (0.9)	2 (1.5)	0 (0)
Joint angles, median (min/max°)^b^			
Inclination angle	8 (− 23/56)	6 (−19/24)^c^	14 (− 23/56)^c^
Version angle	−11 (−55/23)	−13 (− 55/21)^d^	−9 (− 38/23)^d^
Critical shoulder angle	31.5 (17.6/61.6)	29.2 (17.6/40.4)^e^	36.7 (21.9/61.6)^e^

^a^ Measured with the 3D CT

^b^ Measured with the Glenosys automated 3D software planning program

^c^ *P* < 0.001

^d^ *P* = 0.85

^e^ *P* < 0.001

**Table 2:** Preoperative Walch and Sirveaux/Favard Classification distribution of 231 Shoulders from 202 Patients with Osteoarthritis Undergoing Shoulder Replacement

Walch Classification		Sirveaux/Favard Classification	
A1	38 (16,4%)	**E0**	24 (10,4%)
A2	19 (8,2%)		
B1	47 (20,3%)	**E1**	25 (10,8%)
B2	35 (15,1%)		
B3	19 (8,2%)	**E2**	13 (5,6%)
C	3 (1,2%)		
D	3 (1,2%)	**E3**	20 (8,6%)

**Table 3:** Preoperative Fatty Infiltration of Rotator Cuff Muscles according to Goutallier Classification for 231 Shoulders from 202 patients with Osteoarthritis Undergoing Shoulder Replacement

Category	Intact rotator cuff (*N =* 131)					Torn rotator cuff (*N =* 100)				
Goutallier classification, n (%)	**0**	**1**	**2**	**3**	**4**	**0**	**1**	**2**	**3**	**4**
Supraspinatus	1 (0.7)	29 (22.1)	83 (63.3)	16 (12.2)	2 (1.5)	0 (0)	1 (1)	24 (24)	18 (18)	57 (57)
Infraspinatus	1 (0.7)	17 (12.9)	74 (56.4)	34 (25.9)	5 (3.8)	0 (0)	2 (2)	19 (19)	30 (30)	49 (49)
Teres minor	6 (4.5)	62 (47.3)	50 (38.1)	10 (7.6)	3 (2.2)	0 (0)	17 (17)	39 (39)	32 (32)	12 (12)
Subscapularis	6 (4.5)	61 (46.5)	48 (36.6)	15 (11.4)	1 (0.7)	1 (1)	13 (13)	23 (23)	46 (46)	17 (17)

The proportion of torn rotator cuffs was significantly higher in the high-inclination group than in the low-inclination group (61% vs 31%) and in the no inclination group (61% vs 21%); however, the proportions did not differ between the low-inclination and no-inclination groups (31% vs 21%; Table 4). Median glenoid inclination angle and CSA differed significantly between the torn-cuff group and the intact-cuff group in the high-inclination group (Table 4). In the low and no-inclination group, median CSAs, but not median inclination angles differed significantly by rotator cuff status (Table 4). In the high inclination group, 41 of 105 (%39) shoulders had an intact rotator cuff or about 18% of all shoulders.

**Table 4:** Preoperative Characteristics of 231 Shoulders from 202 Patients with Osteoarthritis Undergoing Shoulder Arthroplasty, by Inclination Angle and Rotator Cuff Status

Characteristic	Superior glenoid inclination angle					
	High inclincation (≥ 10°)*n* = 105		Low inclincation (0° to 10°) *n* = 97		No inclincation (≤0°) *n* = 29	
	Intact-cuff	Torn-cuff	Intact-cuff	Torn-cuff	Intact-cuff	Torn-cuff
Shoulders, n (%)	41 (39)	64 (61) ^a^	67 (69.1)	30 (30.9) ^a^	23 (79.3)	6 (20.7) ^a^
Inclination angle, median (min/max), degrees	14 ^b^ (10/24)	14 ^b^ (10/56)	5^c^ (1/9)	6 ^c^ (1/9)	-5^d^ (−18/0)	−3 ^d^ (−23/0)
CSA angle, median (min/max), degrees	31.3 ^e^ (21.5/38.8)	37.8 ^e^ (21.9/61.6)	28.6 ^f^ (17.6/40.4)	32.6 ^f^ (23.9/43.0)	26.9 ^g^ (18.0/31.3)	36.7 ^g^ (27.4/49.7)

*CSA* critical shoulder angle

^a^ Difference between high inclination vs low inclination and no inclination, *P <* 0.001

^b^ Difference between intact-cuff and torn-cuff groups, *P <* 0.001

^c^ Difference between intact-cuff and torn-cuff groups, *P* = 0.114

^d^ Difference between intact-cuff and torn-cuff groups, *P* = 0.518

^e^ Difference between intact-cuff and torn-cuff groups, *P* < 0.023

^f^ Difference between intact-cuff and torn-cuff groups, *P* = 0.002

^g^ Difference between intact-cuff and torn-cuff groups, *P <* 0.001

## Discussion

Measuring glenoid inclination and version angles are critical when planning either anatomic or reverse shoulder arthroplasty. Fortunately, both the version and inclination angles can be measured reliably [17, 20]. The evaluation of the rotator cuff is crucial in determining the type of arthroplasty. Reverse shoulder arthroplasty is mandatory when the rotator cuff is torn. The effects of glenoid version and inclination on rotator cuff integrity are not clearly established. Some recent studies focused on the relationship between rotator cuff integrity, fatty infiltration and glenoid deformity [21–24]. Increased glenoid retroversion was associated with a high cross-sectional area of the supraspinatus and infraspinatus, rather than the low cross-sectional area of subscapularis [22]. Posterior cuff ratio was found to be high in cross-sectional areas of patients with Walch B2 types rather than in Walch A types. Posterior humeral subluxation and glenoid retroversion was as well were related to posterior cuff ratio [21]. Moreover Aleem et al. [21] did not find a correlation of glenoid inclination with rotator cuff muscle area whereas, Walker et al. [24] and Donohue et al. [23] described the association of increased posterior muscle fatty infiltration with posterior glenoid bone loss and glenoid retroversion, which is in line with our findings. Our data showed that posterior and posterosuperior glenoid wear was seen in 98% of patients with rotator cuff tears compared to only 91,5% in rotator cuff intact patients. Unfortunately, the number of patients with rotator cuff intact and significant posterior fatty infiltration were too small for statistical evaluation.

Our values are in line with previously published CT-based studies; the mean (SD) inclination angle was reported as 4.63° (4.86) in non-arthritic cohort [25]. Moreover, in another study, mean (SD) version and inclination angles were found to be − 15.1 (10.6), 8.9 (9.9) [26]. Our results are also similar to those of another study that compared the reliability of measurements made with a 3D software program that reported mean (SD) values of − 9.75 (12.81) for version angle and 7.28 (7.38) for inclination angle in arthritic shoulders [15]. In our study, only a few of shoulders had an inferior inclination angle of less than 0°. Most shoulders in our sample had higher retroversion and superior inclination angles. Moreover, the median version angle we found was the same as that reported by Iannotti and Bercik et al. [13, 14].

Our data showed that in osteoarthritic patients, the CSA was higher in those with secondary osteoarthritis with torn rotator cuffs than in those with intact rotator cuffs and that the CSA was positively correlated with glenoid inclination. Our findings are consistent with those of Moor et al. [8] who reported that a CSA greater than 35° was associated with rotator cuff tears but not with osteoarthritis and that a CSA of less than 30° was associated with osteoarthritis but not rotator cuff tears. Moreover, our results confirm the positive correlation between glenoid inclination and CSA reported by Dagget et al. [5].

In primary arthritis, glenoid wear may be either central or peripheral (mostly posterior), but the association of the direction of glenoid erosion to the deviation of inclination has not been established [4, 27]. In rotator cuff arthropathy, the humeral head generally migrates progressively upwards from the center, leading to superior glenoid erosion [12]. The difference in biomechanics of osteoarthritic shoulders with intact rotator cuffs but different degrees of pre-existing superior or inferior inclination may be related to the individual pattern of muscle forces and the position of the scapula, described as “extrinsic” inclination by Levigne et al. [28] Cases in our group with secondary osteoarthritis with rotator cuff tears, increased superior inclination and posterior-superior glenoid wear pattern, especially with excessive values was related to rotator cuff arthropathy. Nevertheless, in our “intact cuff” group also posterior-superior glenoid wear, but mostly to a minor extend was seen in 35.8% of the patients. In literature this was attributed to rotator cuff muscle atrophy or fatty infiltration [23, 24].

A CT study of patients with arthritic shoulders with B2 glenoid erosion found a different glenoid version angle between the arthritic and normal glenoids in the contralateral shoulder, mostly in the posterior-inferior wear, but no difference in glenoid inclination [27]. However, in our cohort of rotator cuff intact osteoarthritic patients 35.8% of the shoulders had posterior-superior and 55,7% posterior glenoid erosion. Posterior-superior glenoid wear may be related to concomitant rotator cuff muscle atrophy and fatty infiltration even when our rotator cuff intact group was not composed exclusively of B2 classified patients. Moreover, the scatter plots for patients with and without torn-rotator cuffs revealed postero-superior and posterior distribution patterns not only in most of the patients with rotator tears but in those with intact cuffs as well. Our study comprised all arthritic patients rather than only B2 patients, this may be the cause of postero-inferior / postero-superior or posterior erosion disagreement. Some studies have found significant differences in glenoid inclination between shoulders with and without torn rotator cuffs [5, 8], but others have not [9, 29]. Daggett et al. [5] claimed that the degree of glenoid inclination was the crucial factor distinguishing patients with torn rotator cuffs from those with primary osteoarthritis. Favard stated that normal intrinsic glenoid inclination is generally between 0° and 10° [12].

The rate of secondary insufficiency described by Young et al. corresponds well with our high-inclination group with intact rotator cuff. Our high-inclination group had significantly higher proportion of shoulders with rotator cuff tears. Nevertheless, in this group, shoulders with intact rotator cuff were also common; 39% of the high-inclination group; 17.7% of all shoulders. This frequency of preoperative intact rotator cuffs may explain the high incidence of secondary rotator cuff dysfunction in the mid- to long-term follow-up after anatomical shoulder arthroplasty in osteoarthritic shoulders with intact rotator cuffs at time of the index surgery (16.8%; 87/518) as reported in a multicenter study published by Young et al. [7]. Nevertheless, this inference needs the support of clinical data with mid- to long-term follow-up. Favard et al. [12] claimed that the secondary rotator cuff insufficiency seen in patients with an anatomic prosthesis after a long-term follow-up could be related to tendon overload caused by superior translation, which is caused by a superior glenoid inclination angle of 10° or more. Thus, our findings of larger proportions of osteoarthritic shoulders with increased superior inclination and intact rotator cuff are compatible with Young et al. [7] and Favard et al. [12] statements.

### Limitations of the study

Our study has some limitations. Rotator cuff insufficiency was related to the findings on CT scans and operation notes, but were augmented by only 43 MRI examinations, taken only in cases when CT scans were inconclusive. Second, retrospective preoperative data were used for this study. Further studies investigating the clinical outcome after both anatomical and reverse shoulder arthroplasty of patients with superior glenoid inclination exceeding 10 degrees are warranted in order to improve surgical decision making in osteoarthritic patients. Additionally, in rotator cuff intact shoulders with increased superior inclination the influence of minor rotator cuff atrophy and fatty infiltration degrees needs further attention.

## Conclusion

- In rotator cuff deficient patients especially suffering from cuff tear arthropathy superior glenoid erosion is to a larger proportion directed to posterior-superior.
- Based on automated 3D software measurements the majority of patients with secondary osteoarthritic patients with rotator cuff tears show increased superior inclination and wear which is mainly located posterior-superiorly. However, to a considerable amount (35.8%) this finding present as well in rotator cuff intact osteoarthritic shoulders.
- As 18% of all osteoarthritic shoulders with intact rotator cuff had a superior glenoid inclination of more than 10°, future rotator cuff problems might be considered in anatomical shoulder arthroplasty, even when the rotator cuff is intact at the time of surgery.

## Data Availability

Raw data for this study are located and protected at the Rhon Klinikum Campus Bad Neustadt, Department of Shoulder Surgery. The datasets used and/or analyzed during the current study available from the corresponding author on reasonable request.

## Abbreviations

CSA: Critical Shoulder Angle
2D: Two Dimensional
3D: Three Dimensional
CT: Computed Tomography
AP: Antero-posterior
MRI: Magnetic Resonance Imaging

## References

[1] Maurer A, Fucentese SF, Pfirrmann CW, Wirth SH, Djahangiri A, Jost B, Gerber C. Assessment of glenoid inclination on routine clinical radiographs and computed tomography examinations of the shoulder. J Shoulder Elb Surg. 2012. 10.1016/j.jse.2011.07.010. 10.1016/j.jse.2011.07.010 22036540

[2] Nyffeler RW, Meyer DC. Acromion and glenoid shape: why are they important predictive factors for the future of our shoulders? EFORT Open Rev. 2017. 10.1302/2058-5241.2.160076. 10.1302/2058-5241.2.160076 PMC5467673 28630752

[3] Strauss EJ, Roche C, Flurin PH, Wright T, Zuckerman JD. The glenoid in shoulder arthroplasty. J Shoulder Elb Surg. 2009. 10.1016/j.jse.2009.05.008. 10.1016/j.jse.2009.05.008 19574062

[4] Walch G, Badet R, Boulahia A, Khoury A (1999). Morphologic study of the glenoid in primary glenohumeral osteoarthritis. J Arthroplast.

[5] Daggett M, Werner B, Collin P, Gauci MO, Chaoui J, Walch G. Correlation between glenoid inclination and critical shoulder angle: a radiographic and computed tomography study. J Shoulder Elb Surg. 2015. 10.1016/j.jse.2015.07.013. 10.1016/j.jse.2015.07.013 26350880

[6] Terrier A, Merlini F, Pioletti DP, Farron A. Total shoulder arthroplasty: downward inclination of the glenoid component to balance supraspinatus deficiency. J Shoulder Elb Surg. 2009. 10.1016/j.jse.2008.11.008. 10.1016/j.jse.2008.11.008 19243979

[7] Young AA, Walch G, Pape G, Gohlke F, Favard L. Secondary rotator cuff dysfunction following total shoulder arthroplasty for primary glenohumeral osteoarthritis: results of a multicenter study with more than five years of follow-up. J Bone Joint Surg Am. 2012. 10.2106/JBJS.J.00727. 10.2106/JBJS.J.00727 22419408

[8] Moor BK, Bouaicha S, Rothenfluh DA, Sukthankar A, Gerber C. Is there an association between the individual anatomy of the scapula and the development of rotator cuff tears or osteoarthritis of the glenohumeral joint? A radiological study of the critical shoulder angle. Bone and Joint Journal. 2013. 10.1302/0301-620X.95B7. 10.1302/0301-620X.95B7.31028 23814246

[9] Bishop JL, Kline SK, Aalderink KJ, Zauel R, Bey MJ. Glenoid inclination: in vivo measures in rotator cuff tear patients and associations with superior glenohumeral joint translation. J Shoulder Elb Surg. 2009. 10.1016/j.jse.2008.08.002. 10.1016/j.jse.2008.08.002 PMC2669899 19062313

[10] Hughes RE, Bryant CR, Hall JM, Wening J, Huston LJ, Kuhn JE, Carpenter JE, Blasier RB. Glenoid inclination is associated with full-thickness rotator cuff tears. Clin Orthop Relat Res. 2003. 10.1097/01.blo.0000043055.62337.a8. 10.1097/00003086-200302000-00016 12567135

[11] Sirveaux F, Favard L, Oudet D, Huquet D, Walch G, Molé D. Grammont inverted total shoulder arthroplasty in the treatment of glenohumeral osteoarthritis with massive rupture of the cuff. J Bone Joint Surg. 2004. 10.1302/0301-620X.86B3. 10.1302/0301-620x.86b3.14024 15125127

[12] Favard L, Berhouet J, Walch G, Chaoui J, Levigne C. Superior glenoid inclination and glenoid bone loss: definition, assessment, biomechanical consequences, and surgical options. Orthopade. 2017. 10.1007/s00132-017-3496-1. 10.1007/s00132-017-3496-1 29098355

[13] Bercik MJ, Kruse K 2nd, Yalizis M, Gauci MO, Chaoui J, Walch G. A modification to the Walch classification of the glenoid in primary glenohumeral osteoarthritis using three-dimensional imaging. J Shoulder Elb Surg. 2016. 10.1016/j.jse.2016.03.010. 10.1016/j.jse.2016.03.010 27282738

[14] Iannotti JP, Jun BJ, Patterson TE, Ricchetti ET. Quantitative measurement of osseous pathology in advanced Glenohumeral osteoarthritis. J Bone Joint Surg Am. 2017. 10.2106/JBJS.16.00869. 10.2106/JBJS.16.00869 28872528

[15] Boileau P, Cheval D, Gauci MO, Holzer N, Chaoui J, Walch G. Automated three-dimensional measurement of Glenoid version and inclination in arthritic shoulders. J Bone Joint Surg Am. 2018. 10.2106/JBJS.16.01122. 10.2106/JBJS.16.01122 29298261

[16] Iannotti JP, Weiner S, Rodriguez E, Subhas N, Patterson TE, Jun BJ, Ricchetti ET. Three-dimensional imaging and templating improve glenoid implant positioning. J Bone Joint Surg Am. 2015. 10.2106/JBJS.N.00493. 10.2106/JBJS.N.00493 25878309

[17] Moineau G, Levigne C, Boileau P, Young A, Walch G (2012). French Society for S, elbow: three-dimensional measurement method of arthritic glenoid cavity morphology: feasibility and reproducibility. Orthop Traumatol Surg Res.

[18] Goutallier D, Postel JM, Bernageau J, Lavau L, Voisin M-C (1994). Fatty muscle degeneration in cuff ruptures pre- and postoperative evaluation by CT scan. Clin Orthop Relat Res.

[19] Hamada K, Fukuda H, Mikasa M, Kobayashi Y (1990). Roentgenographic findings in massive rotator cuff tears. A long-term observation. Clin Orthop Relat Res.

[20] Lewis GS, Armstrong AD. Glenoid spherical orientation and version. J Shoulder Elb Surg. 2011. 10.1016/j.jse.2010.05.012. 10.1016/j.jse.2010.05.012 20932782

[21] Aleem AW, Chalmers PN, Bechtold D, Khan AZ, Tashjian RZ, Keener JD. Association between rotator cuff muscle size and Glenoid deformity in primary Glenohumeral osteoarthritis. J Bone Joint Surg Am. 2019. 10.2106/JBJS.19.00086. 10.2106/JBJS.19.00086 31567672

[22] Chalmers PN, Beck L, Miller M, Stertz I, Henninger HB, Tashjian RZ. Glenoid retroversion associates with asymmetric rotator cuff muscle atrophy in those with Walch B-type Glenohumeral osteoarthritis. J Am Acad Orthop Surg. 2019. 10.5435/JAAOS-D-18-00830. 10.5435/JAAOS-D-18-00830 PMC7064422 31517880

[23] Donohue KW, Ricchetti ET, Ho JC, Iannotti JP. The association between rotator cuff muscle fatty infiltration and Glenoid morphology in Glenohumeral osteoarthritis. J Bone Joint Surg Am. 2018. 10.2106/JBJS.17.00232. 10.2106/JBJS.17.00232 29509615

[24] Walker KE, Simcock XC, Jun BJ, Iannotti JP, Ricchetti ET. Progression of Glenoid morphology in Glenohumeral osteoarthritis. J Bone Joint Surg Am. 2018. 10.2106/JBJS.17.00064. 10.2106/JBJS.17.00064 29298260

[25] Ghafurian S, Galdi B, Bastian S, Tan V, Li K. Computerized 3D morphological analysis of glenoid orientation. J Orthop Res. 2016. 10.1002/jor.23053. 10.1002/jor.23053 26400654

[26] Werner BS, Hudek R, Burkhart KJ, Gohlke F. The influence of three-dimensional planning on decision-making in total shoulder arthroplasty. J Shoulder Elb Surg. 2017. 10.1016/j.jse.2017.01.006. 10.1016/j.jse.2017.01.006 28162884

[27] Knowles NK, Ferreira LM, Athwal GS. Premorbid retroversion is significantly greater in type B2 glenoids. J Shoulder Elb Surg. 2016. 10.1016/j.jse.2015.11.002. 10.1016/j.jse.2015.11.002 26895600

[28] Levigne C, Garret J, Boileau P, Alami G, Favard L, Walch G. Scapular notching in reverse shoulder arthroplasty: is it important to avoid it and how? Clin Orthop Relat Res. 2011. 10.1007/s11999-010-1695-8. 10.1007/s11999-010-1695-8 PMC3148391 21116754

[29] Kandemir U, Allaire RB, Jolly JT, Debski RE, McMahon PJ. The relationship between the orientation of the glenoid and tears of the rotator cuff. J Bone Joint Surg (Br). 2006. 10.1302/0301-620X.88B8. 10.1302/0301-620X.88B8.17732 16877616

